# Analysis of Comfort during Transfer by a Dual-Arm Care Robot Based on Human Body Pressure and Surface Electromyographic Signals

**DOI:** 10.3390/bioengineering10080903

**Published:** 2023-07-30

**Authors:** Qifei Guan, Zhiqiang Yang, Hao Lu, Shijie Guo

**Affiliations:** 1 Academy for Engineering and Technology, Fudan University, Shanghai 200433, China; 2 Shanghai Engineering Research Center of AI & Robotics, Shanghai 200433, China; 3 Engineering Research Center of AI & Robotics, Ministry of Education, Shanghai 200433, China; 4 College of Electronic Information and Automation, Tianjin University of Science and Technology, Tianjin 300222, China

**Keywords:** care robots, human–robot biomechanical modeling, comfort evaluation, surface electromyography

## Abstract

In-home elderly care faces a crucial challenge regarding mobility among essential assistive devices, for which dual-arm care robots present a viable solution. However, ensuring human comfort in human–robot interactions necessitate quantifiable standards. Currently, the field lacks accurate biomechanical model solutions and objective comfort evaluation. In response to this need, this study proposes a method for solving human–robot statics models based on real-time pressure and position information. Employing the Optitrack motion capture system and Tekscan pressure sensors, we collect real-time positional and pressure data. This information is then incorporated into our human–robot statics model, facilitating the instantaneous calculation of forces and moments within the human body’s sagittal plane. Building on this, comprehensive research literature review and meticulous questionnaire surveys are conducted to establish a comprehensive comfort evaluation function. To validate this function, experiments are performed to enable real-time assessment of comfort levels experienced during the process of transferring the human body. Additionally, the Noraxon surface electromyography (sEMG) sensors are utilized to capture real-time sEMG signals from the erector spinae, adductor muscles and quadratus lumborum, thereby providing objective validation for the comfort evaluation function. The experimental findings demonstrate that the proposed methodology for evaluating comfort achieves an accuracy rate of 85.1%.

## 1. Introduction

With the escalating global phenomenon of population aging, the number of individuals with disabilities in the elderly cohort is undergoing a rapid surge. In 2021, China alone had 44 million disabled elderly individuals [1]. Moreover, projections indicate a continuous increase in the population of disabled elderly individuals in China over the next decade, with an estimated figure of 77 million by 2030 [2]. The scarcity of caregiving personnel has emerged as a pertinent societal challenge [3].

In the realm of caregiving, enabling the seamless mobility of disabled elderly individuals among essential assistive devices not only presents the most physically demanding aspect for caregivers but also represents the core predicament constraining in-home elderly care [4,5]. The advent of dual-arm care robots has emerged as a viable panacea to address this predicament, as they effectively facilitate the transfer and transportation of disabled elderly individuals to designated locations, thereby alleviating the workload burden on caregivers. However, this innovation poses heightened requisites for ensuring optimal comfort levels throughout the process of transferring and transporting the human body [6]. Consequently, the objective assessment of human comfort has become an exigent issue that necessitates immediate attention.

Presently, several scholars have conducted research on the issue of human comfort in various postures. For investigating comfort in static body postures, Li conducted a study on the comfort experienced by individuals on different seat cushions, employing objective metrics such as stress distribution and average sitting pressure, along with subjective comfort rating scales [7]. Liu analyzed indicators such as seat contact area and maximum sitting pressure to examine the relationship between human comfort and seat cushion shape, incorporating subjective evaluation methods [8]. Xiong evaluated lumbar muscle fatigue by assessing sEMG signals from the erector spinae muscles. They investigated the influence of seated posture on human comfort during aviation, employing subjective evaluation methods [9]. Anjani employed a Statistical Package for the Social Sciences (SPSS) analysis of subjective questionnaires to study the impact of seat spacing on human comfort and applied significance analysis to rank the magnitude of factors influencing human comfort [10]. Naddeo assessed the influence of different spinal postures on human comfort using subjective questionnaire surveys and a method to weigh the effect of the perceived spinal discomfort on overall postural comfort was proposed [11]. Liu analyzed upper limb muscle fatigue by measuring sEMG signals from upper limb muscles and performed an analysis of human comfort through subjective questionnaires [12]. In terms of studying the overall process of transferring and transporting the human body, Ding established a comfort evaluation function by formulating a human force balance equation and measuring sEMG signals during the transfer process. They determined the parameter values of the comfort evaluation function through questionnaire surveys [13]. Liu examined human comfort by developing a human–robot biomechanical model and conducting ADAMS simulations. They validated the comfort experienced during the transfer and transportation process through experimental investigations and subjective questionnaire surveys [14].

These studies have provided valuable insights into evaluating human comfort in various scenarios. However, existing research on the comfort of human posture only focuses on analyzing comfort at fixed positions on the body, overlooking the study of comfort during dynamic changes in body posture. Moreover, when objectively assessing human comfort, the employed evaluation metrics are limited to single indicators such as pressure distribution or surface electromyography signals, lacking comprehensive validation of comfort assessment. Within the domain of holistic comfort assessment methods, two significant issues prevail. Firstly, in the computation of comfort evaluation functions, determining the magnitudes of forces and moments exerted on the human body poses an intractable problem, necessitating assumptions and additional conditions to supplement the equations in the system. However, this approach introduces errors in the calculation results while simultaneously estimating force and moment magnitudes. Secondly, the validation of comfort evaluation functions is primarily reliant on subjective questionnaires, thereby lacking objective verification. Although subjective assessment methods constitute an essential aspect of comfort evaluation, their inherent subjectivity introduces randomness, resulting in uncertain validation outcomes.

This paper addresses the issue of indeterminacy in human–machine biomechanical models and proposes a real-time method for resolving the challenge of determining the forces acting on the human body in the sagittal plane. Experimental validation is conducted to verify the proposed approach. The method employs Tekscan pressure sensors and an Optitrack motion capture system to acquire real-time pressure and angular data of the human body. These data are then utilized as inputs into a static human–machine system model, enabling the determination of frictional forces and joint torques experienced by the body. Simultaneously, a real-time method for evaluating human comfort is established and validated using sEMG signals. A comfort evaluation function is developed through questionnaire surveys and the proposed method for determining human forces is utilized to calculate real-time values of this function, thereby providing a quantitative basis for comfort assessment. Additionally, Noraxon surface electromyography sensors are employed to capture real-time surface electromyography information, which is combined with subjective questionnaire responses to validate the comfort evaluation method. Experimental results demonstrate an 85.1% accuracy rate for the proposed comfort evaluation method.

## 2. Materials and Methods

### 2.1. Analysis of Human Motion and Development of Human–Machine Statics Model

During the process of transfer and transportation, human movement can be segmented into distinct phases, including the lifting phase, posture adjustment phase, horizontal displacement phase and lowering phase. Variations in human comfort are significantly pronounced across these different phases, necessitating a comprehensive analysis of human motion during transfer and transportation. Moreover, the comfort experienced by individuals is closely linked to the magnitude of forces and moments exerted on the body. Therefore, prior to establishing a comfort evaluation function, it is imperative to accurately resolve the contact forces between the human body and the robot, thereby facilitating the development of a precise human–machine biomechanical model. Additionally, given the relatively slow movement of the robotic arm during transfer and transportation, it is reasonable to disregard the dynamic aspects and instead focus on constructing a human–machine statics model.

#### 2.1.1. Analysis of Human Motion

During the process of transferring care subjects from one bed to another with an assistive device using dual-arm care robots, various changes occur in the care recipient’s posture. In the lifting phase, the care subject maintains an initial position while the robotic arms extend between the human body and the nursing bed, gradually lifting the individual. At the moment of lifting, the point of force application transitions from the hips to the back and legs of the care subject. Throughout the lifting process, there is a downward sliding tendency of the body and the muscles near the hip joint become tense and exert force to prevent slippage. The magnitude of force required by the body is determined by the arm distance of the care robot and the coefficient of friction between the human body and the robotic interface. A greater arm distance or a lower friction coefficient necessitates a higher force to sustain the body posture, whereas a smaller arm distance or a higher friction coefficient requires less force to maintain the desired posture.

During the stages of posture adjustment and planar displacement, the care robot adjusts the human body posture by controlling the waist joint or the position of the robotic arm. During this phase, the body undergoes a transition from a supine position to a relatively comfortable posture while also experiencing planar displacement. In the lowering phase, the care subject’s waist remains stationary, while the care robot adjusts the arm’s position to align the body precisely above the nursing bed or other relevant assistive device. Subsequently, the robotic arm’s relative height is fine-tuned to modify the body’s posture and a gradual lowering of the care subject ensues. Once the hips of the individual make contact with the assistive device, the robotic arm retracts in a controlled manner, successfully completing the transfer and transportation task.

From the aforementioned analysis, it is evident that the most significant changes in force and torque on the human body occur during the stages of posture adjustment as well as during the lifting and lowering phases. Therefore, it is imperative to concentrate on conducting focused research regarding the impact of posture modifications on the variations in human comfort.

#### 2.1.2. Development of Human–Robot Statics Model

**Figure 1:**
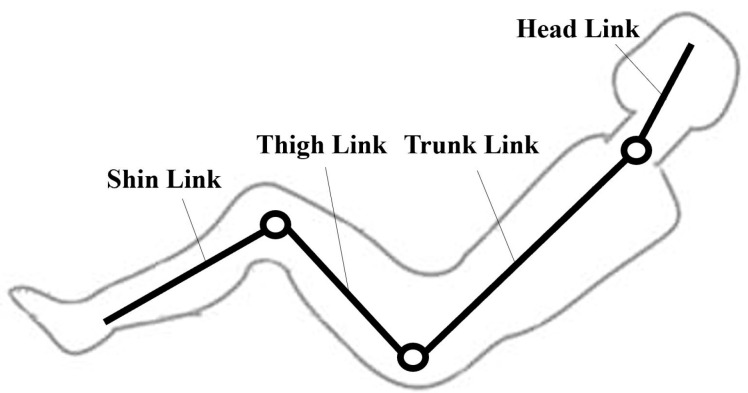
Simplified four-link model of the human body.

To facilitate the analysis of human–robot interaction forces, it is advantageous to simplify the human body to the sagittal plane due to its inherent bilateral symmetry. Consequently, the human body needs to be initially approximated using a simplified linkage model. Commonly employed models include the two-link model [15], three-link model [16], four-link model [17] and six-link model [18]. During the transfer and transportation process, the primary force-bearing joints of the human body are the hip joint, knee joint and cervical spine. Based on these joint divisions, the human body can be simplified into a four-link model comprising the shin, thigh, trunk and head. The simplified four-link model is depicted in Figure 1.

**Figure 2:**
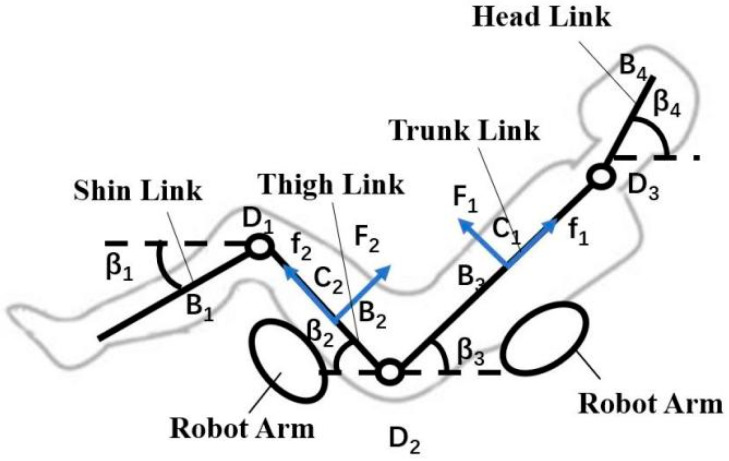
Statics model of the human–robot system.

In the process of transfer and transportation, the interaction forces between the human body and the robotic arm are predominantly localized at the forearm of the robotic arm. Thus, it is feasible to simplify the contact between the robotic arm and the human body as a planar interface. Consequently, the four-link model mentioned earlier is employed for the establishment of a human–robot statics model. The forces acting on the human body include both external forces and internal forces. During the transfer and transportation process, the external forces experienced by the human body consist of pressures *F_1_* and *F_2_* exerted on the thigh and back regions, respectively, in addition to the friction forces *f_1_* and *f_2_*. Meanwhile, the internal forces arise as joint moments *M_1_*, *M_2_* and *M_3_* generated within the human body to maintain equilibrium. The force distribution on the human body is depicted in Figure 2.

In Figure 2, the variables *D_1_*, *D_2_* and *D_3_* correspond to the knee joint, hip joint and neck joint, respectively. The human body is partitioned into four interconnected segments: shin, thigh, trunk and head. The positions of *B_1_*, *B_2_*, *B_3_* and *B_4_* represent the centroids of these four segments, while *C_1_* and *C_2_* indicate the contact locations between the robotic arm and the human body. The angles *β_1_*, *β_2_*, *β_3_* and *β_4_* represent the orientations of the shin, thigh, trunk and head, respectively, with respect to the horizontal axis. The variables *F_1_* and *f_1_* refer to the applied pressure and frictional force acting on the human back, while *F_2_* and *f_2_* represent the pressure and frictional force experienced by the legs.

Given the unequal mass distribution and varying centroid positions among the different body segments, meticulous calculations are required for each simplified link of the human body model. To maintain consistency with prevailing practices, the Brawne–Fisher model is selected for this study and its corresponding parameters are outlined in Table 1. In Table 1, the center of gravity radius denotes the ratio of the distance between the center of gravity and the upper joint’s center to the longitudinal length of the corresponding body segment in an upright stance. The symbol “#” signifies the intersection point between the upper edges of the ears and the mid-sagittal plane, serving as an indicator of the head’s centroid position. Similarly, the symbol “##” designates the joint between the middle finger and the palm, representing the centroid position of the hand.

After simplifying the human body into a four-link model, the trunk, upper arm, forearm and hand are combined into the trunk link, while the shin and foot form the shin link. Therefore, the synthesized parameters for the four-link segments are presented in Table 2.

Due to the slow movement of the robotic arm, the human body can be approximated as being in a state of force equilibrium. Consequently, force equilibrium equations can be formulated in the X and Y directions, as presented in Equations (1) and (2), respectively.

(1) $$f_{1}cos\beta_{3}-F_{1}sin\beta_{3}=f_{2}cos\beta_{2}-F_{2}sin\beta_{2}$$

(2) $$f_{1}sin\beta_{3}+F_{1}cos\beta_{3}=G-f_{2}sin\beta_{2}-F_{2}cos\beta_{2}$$

Furthermore, considering that the torque generated by external forces is negligible, a torque equilibrium equation can be established at the knee joint, as demonstrated by Equation (3).

(3) $$F_{2}\left[\bar{C_{1}D_{2}}+\bar{C_{2}D_{2}}\cos\left(\beta_{2}+\beta_{3}\right)\right]+f_{1}\bar{C_{2}D_{2}}\sin\left(\beta_{2}+\beta_{3}\right)=G(x_{G}-x_{D1})$$

Regarding the joint torques exerted by the human body to maintain its own posture, the knee joint torque *M_1_* can be computed by evaluating the product of the gravitational force acting on the shin and its corresponding lever arm, as outlined in Equation (4).

(4) $$M_{1}=G_{1}\times \bar{D_{1}B_{1}}\times cos\beta_{1}$$

Similarly, the hip joint torque *M_2_* and neck joint torque *M_3_* can be expressed through Equations (5) and (6), respectively.

(5) $$M_{2}=G_{1}\times \left(\bar{D_{1}D_{2}}\times cos\beta_{2}+\bar{D_{1}B_{1}}\times cos\beta_{1}\right)+G_{2}\times \bar{D_{2}B_{2}}\times cos\beta_{2}-F_{2}l_{2}$$

(6) $$M_{3}=G_{4}\times \bar{D_{3}B_{4}}\times cos\beta_{4}$$

Herein, *M_1_*, *M_2_* and *M_3_* denote the internal joint torques at the knee, hip and neck joints, respectively. *G_1_*, *G_2_*, *G_4_* and *G* represent the weights of the shin, thigh, head and total body mass, respectively, while *X_G_* represents the horizontal coordinate of the body’s center of gravity.

At this stage, the system of equations comprises a total of seven unknowns, namely *F_1_*, *F_2_*, *f_1_*, *f_2_*, *M_1_*, *M_2_* and *M_3_*, while the available number of equations remains limited to six. Therefore, it is imperative to augment the equation system. However, due to the static nature of the frictional force encountered, its precise value remains indeterminate and poses challenges in measurement. Consequently, establishing a definitive relationship between the frictional force and the pressure exerted on the human body proves unattainable. Thus, it becomes essential to utilize additional known information to solve the equations and ascertain a conclusive solution for the equation system.

### 2.2. Human–Robot Statics Model Solution and Comfort Evaluation

#### 2.2.1. Solution of the Human–Robot Statics Model

The equation system of the human–robot statics model comprises seven unknowns while having only six equations. Therefore, it necessitates the addition of a known quantity or an extra equation. Conventional research approaches have posited a dependence between frictional force and normal pressure, thus supplementing an equation that establishes the relationship between frictional force and normal pressure to solve the human–robot statics model. However, in the process of transfer and transportation, the frictional force between the human and the robot is characterized by static friction, rendering the determination of its specific value and its relationship with pressure unfeasible. Consequently, existing research methodologies may lead to inaccuracies when solving for forces and moments. To circumvent this issue and enhance the precision of the human–robot statics model, this study adopts a real-time pressure acquisition method by supplementing real pressure values to compute forces and moments, specifically by employing a Tekscan pressure sensor for real-time capture of pressure between the back and the robotic arm.

Each Tekscan pressure sensor comprises two polyester film pieces, with each piece housing 32 rows of conductive strips [20]. When the two polyester film pieces are vertically assembled, the intersection points on the films serve as pressure sensing points. During operation, when the pressure is at zero, the resistance at the sensing points reaches its maximum. As pressure is applied, the resistance at the sensing points proportionally decreases with increasing pressure. Thus, the resistance values at each sensing point can be measured via scanning circuits, providing real-time feedback on the pressure information at each sensing point.

Therefore, before acquiring real-time pressure information, calibration of the pressure sensors is necessary. By vertically placing objects of known different weights on the pressure sensor and recording the corresponding pressure data, the relationship between pressure information and the acquired electrical signals can be established, enabling accurate pressure values to be determined. Due to the substantial data collection of the pressure sensors and the relatively slow movement of the bimanual care robot, pressure data is sampled at a rate of 20 frames per second. After merging and processing the pressure data, real-time pressure values can be obtained.

To obtain real-time internal moments and frictional forces in the human–robot statics model, real-time acquisition of body position information is required. This includes the angles *β_1_*, *β_2_*, *β_3_* and *β_4_* between the shin, thigh, trunk and head, respectively, and the horizontal plane. This information is obtained through the Optitrack motion capture system.

The Optitrack system is an optical motion capture system comprising cameras equipped with infrared light emitters, a switch, heavy-duty tripods, marker labels, calibration rods, computer software, and other components. It can collect data from up to six cameras at a rate of 120 frames per second, and real-time data recording and display are available through the software. To correspond with the pressure information and surface electromyography data, the position data is collected at a frequency of 100 frames per second in this study. The main principle of the Optitrack motion capture system involves determining the position of marker points using the common field of view of different cameras. By attaching marker points to the human body and calibrating the appropriate camera positions beforehand, real-time human body pose information can be calculated. Thus, prior to system usage, calibration of camera positions is conducted using calibration rods.

Through the continuous movement of three fixed marker points on the calibration rod, the system continuously captures these marker points and analyzes the relative positions of each motion capture camera. To ensure the accuracy of camera position information, each camera should scan more than 4000 marker points. Subsequently, a horizontal calibration device was utilized to assess the positional relationship between the cameras and the horizontal plane, thereby determining the precise location of each motion capture camera. Consequently, the captured marker point positions are accurate.

The specific angle calculation formula is as follows. The angle of the shin can be represented by the positional data of the ankle joint *A_1_* and the knee joint *A_2_*, denoted as *X_1_*, *Y_1_*, *Z_1_*, *X_2_*, *Y_2_* and *Z_2_*, as illustrated in Equation (7). The *B_x_* point and the corresponding *A_x_* point lie on the same horizontal line.

(7) $$\beta_{1}=arccos\frac{\vec{A_{2}A_{1}}\cdot \vec{A_{2}B_{2}}}{\left|\vec{A_{2}A_{1}}\right|\left|\vec{A_{2}B_{2}}\right|}=arccos\frac{{\left(x_{1}-x_{2}\right)}^{2}+{\left(y_{1}-y_{2}\right)}^{2}}{\sqrt{{\left(x_{1}-x_{2}\right)}^{2}+{\left(y_{1}-y_{2}\right)}^{2}+{\left(z_{1}-z_{2}\right)}^{2}}\sqrt{{\left(x_{1}-x_{2}\right)}^{2}+{\left(y_{1}-y_{2}\right)}^{2}}}$$

**Figure 3:**
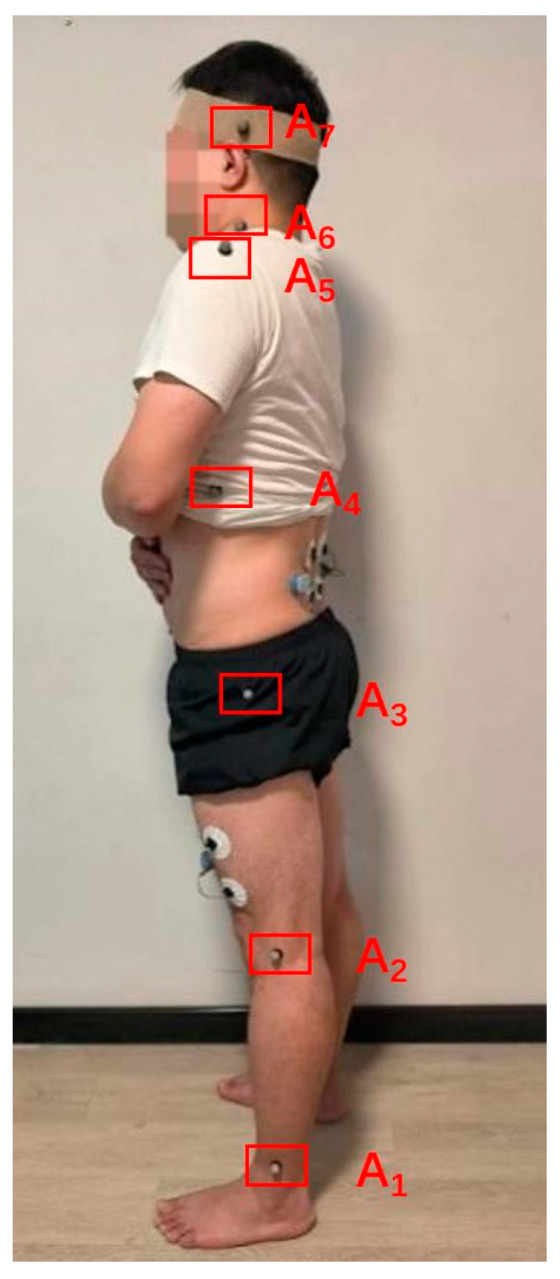
Marker point placement.

Similarly, to obtain the remaining angle data, it is necessary to affix marker points at seven key positions: ankle joint *A_1_*, knee joint *A_2_*, hip joint *A_3_*, trunk *A_4_*, shoulder joint *A_5_*, neck joint *A_6_* and head *A_7_*, as illustrated in Figure 3.

Similarly, angles *β_2_*, *β_3_* and *β_4_* can be calculated using Equations (8)–(10).

(8) $$\beta_{2}=arccos\frac{{\left(x_{2}-x_{3}\right)}^{2}+{\left(y_{2}-y_{3}\right)}^{2}}{\sqrt{{\left(x_{2}-x_{3}\right)}^{2}+{\left(y_{2}-y_{3}\right)}^{2}+{\left(z_{2}-z_{3}\right)}^{2}}\sqrt{{\left(x_{2}-x_{3}\right)}^{2}+{\left(y_{2}-y_{3}\right)}^{2}}}$$

(9) $$\beta_{3}=arccos\frac{{\left(x_{4}-x_{3}\right)}^{2}+{\left(y_{4}-y_{3}\right)}^{2}}{\sqrt{{\left(x_{4}-x_{3}\right)}^{2}+{\left(y_{4}-y_{3}\right)}^{2}+{\left(z_{4}-z_{3}\right)}^{2}}\sqrt{{\left(x_{4}-x_{3}\right)}^{2}+{\left(y_{4}-y_{3}\right)}^{2}}}$$

(10) $$\beta_{4}=arccos\frac{{\left(x_{7}-x_{6}\right)}^{2}+{\left(y_{7}-y_{6}\right)}^{2}}{\sqrt{{\left(x_{7}-x_{6}\right)}^{2}+{\left(y_{7}-y_{6}\right)}^{2}+{\left(z_{7}-z_{6}\right)}^{2}}\sqrt{{\left(x_{7}-x_{6}\right)}^{2}+{\left(y_{7}-y_{6}\right)}^{2}}}$$

In the aforementioned approach, *β_1_*, *β_2_*, *β_3_*, *β_4_* and *F_1_* are known quantities. After incorporating the joint position information and back pressure data into the system of equations, the equation set consists of six unknowns (*F_2_*, *f_1_*, *f_2_*, *M_1_*, *M_2_* and *M_3_*) and six mechanical equations. As a result, the human–machine statics model becomes a solvable problem. Upon solving the model, the explicit parameters of the human body, such as the pressures and friction forces exerted on the leg and back (*F_1_*, *F_2_*, *f_1_* and *f_2_*) and the internal joint moments at the knee, hip and neck (*M_1_*, *M_2_* and *M_3_*), are obtained, providing a basis for the establishment of comfort evaluation.

#### 2.2.2. Establishment of a Comfort Evaluation Function

To evaluate the comfort of the human body during the transfer and transport process, it is necessary to establish a comfort evaluation function. Since the establishment of an evaluation function requires determining parameters, the factors influencing human comfort are first analyzed.

Through a review of existing research on comfort evaluation [7,8,13], it is evident that the selected indicators primarily pertain to the external forces applied to the human body and the internal joint moments. Analyzing these indicators reveals that human comfort is mainly influenced by the magnitude of pressure, frictional forces, and joint moments. In a singular human–robot interaction scenario, where the contact area between humans and robots remains relatively constant, the pressure magnitude can effectively represent the intensity of pressure. Consequently, comfort evaluation research typically focuses on studying pressure, frictional forces, and joint moments. Moreover, after determining the evaluation indicators, the calculation methodology for the comfort evaluation function should be established. By consulting relevant literature on the comfort evaluation of care robot transfer and transport [13,19], the general form of the comfort evaluation function is obtained as expressed in Equation (11).

In the human–machine system statics model, the main factors influencing human comfort are the pressures and friction forces (*F_1_*, *F_2_*, *f_1_* and *f_2_*) applied to the leg and back by the robotic arm, as well as the moments (*M_1_*, *M_2_* and *M_3_*) exerted on the knee, hip and neck joints. At each position, for each set of forces and moments, there corresponds a corresponding comfort evaluation. Therefore, the comfort evaluation value can be regarded as a function based on the independent variables *F_1_*, *F_2_*, *f_1_*, *f_2_*, *M_1_*, *M_2_* and *M_3_*. When the magnitudes of the forces and moments are smaller, the comfort evaluation value is smaller, indicating greater comfort for the human body. Conversely, when the magnitudes of the forces and moments are larger, the comfort evaluation value is larger, indicating greater discomfort for the human body.

Furthermore, under the same magnitudes of forces and moments, each variable has a different impact on comfort. For example, due to the distribution of body mass, the weight of the back is significantly higher than that of the legs, resulting in higher forces on the back compared to the legs. Therefore, when the forces on the back and legs are equal, the legs are comparatively less comfortable than the back. Hence, the comfort evaluation function cannot be simply considered as the summation of individual forces and moments; it needs to take into account the values of each force under the least comfortable conditions and then express the value of that force as its actual value divided by its maximum value.

Furthermore, the impact of individual forces and moments on human comfort varies. For instance, the knee joint moment has a lesser effect on human comfort compared to the moments exerted on the hip joint and neck joint. Therefore, it is necessary to adjust the representation of each force using appropriate parameters. Subsequently, after defining the evaluation criteria, we determined the computation approach for the comfort evaluation function, which is generally represented by Equation (11), based on the relevant literature on comfort evaluation during human transfer and transportation using care robots [13,19].

(11) $$s=\omega_{1}{\left(\left|\frac{F_{1}}{F_{1max}}\right|\right)}^{n1}+\omega_{2}{\left(\left|\frac{F_{2}}{F_{2max}}\right|\right)}^{n2}+\omega_{3}{\left(\left|\frac{f_{1}}{f_{1max}}\right|\right)}^{n3}+\omega_{4}{\left(\left|\frac{f_{2}}{f_{2max}}\right|\right)}^{n4}+\omega_{5}{\left(\left|\frac{M_{1}}{M_{1max}}\right|\right)}^{n5}+\omega_{6}{\left(\left|\frac{M_{2}}{M_{2max}}\right|\right)}^{n6}+\omega_{7}{\left(\left|\frac{M_{3}}{M_{3max}}\right|\right)}^{n7}$$

In the equation, *ω_1_*, …, *ω_7_* represent the coefficients of each evaluation item, and *n_1_*, …, *n_7_* represent the exponents of each evaluation item. *F_1max_*, *F_2max_*, *f_1max_*, and *f_2max_* denote the maximum values of the corresponding forces obtained from the real-time information of the current set. *M_1max_*, *M_2max_* and *M_3max_* represent the torque values calculated when the cosine values of each angle are equal to 1.

Regarding the determination of the function’s parameters, as comfort evaluation is subjective and based on human perception, we employed subjective assessment questionnaires to establish these parameters. Each parameter uniquely influences a specific aspect, and higher parameter values result in higher comfort evaluation function values.

### 2.3. Validation Experiment

**Figure 4:**
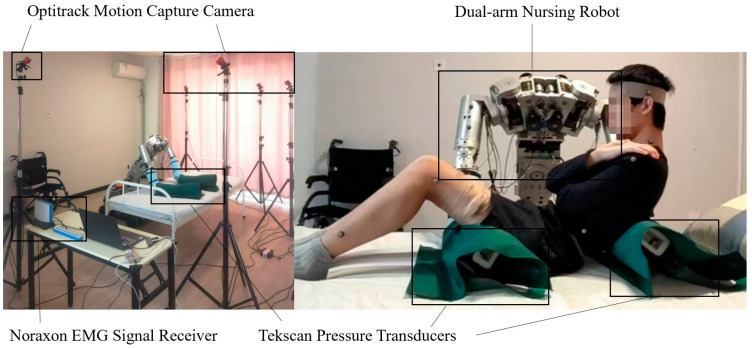
Dual-arm care robot embrace experiment platform.

To validate the real-time solving method of the human–machine statics model and the human body comfort evaluation function, a dual-arm care robot platform was utilized in this experiment. The mechanical arm of the platform has six degrees of freedom, allowing for the adjustment of human body postures for transfer and handling experiments in various positions. During the adjustment of human body postures, real-time information of body posture and pressure was acquired using the Optitrack motion capture system and Tekscan pressure sensors, which were then incorporated into the human–machine statics model for solving. Subsequently, the real-time solved posture and pressure information were input into the comfort evaluation function to obtain the value of the comfort evaluation. Additionally, surface electromyography information was collected in real-time using Noraxon sEMG sensors to further validate the comfort evaluation function. The experimental setup of the dual-arm care robot for human embrace is illustrated in Figure 4.

A total of seven healthy adult subjects were recruited for this experiment, with ages ranging from 19 to 30 years old and an average age of 25 years. The subjects’ heights ranged from 159 cm to 183 cm, and weights ranged from 49 kg to 61 kg. The specific details are presented in Table 3. Prior to each experiment, the length of each segment of the subjects’ bodies was measured in advance for data analysis.

During the scene setup phase, the Optitrack motion capture sensor system was calibrated using calibration rods, and the Tekscan pressure sensors were placed on the robotic arm and calibrated for pressure measurement. Simultaneously, the Noraxon sEMG sensor system was tested to ensure proper acquisition of sEMG signals.

**Figure 5:**
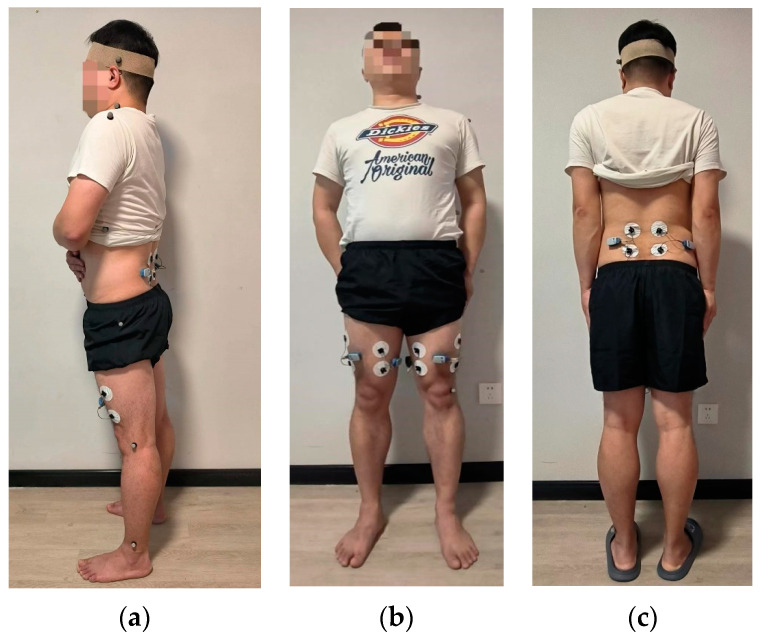
Marker placement and electrode placement: (**a**) Marker placement (**b**) Adductor and tensor fasciae latae electrode placement (**c**) Erector spinae electrode placement.

In the preparation phase of the experiment, reflective markers were attached to seven key positions on the human body, including the ankle joint, knee joint, hip joint, trunk, shoulder joint, neck joint, and head, as well as on the robotic arm to determine the contact points between the arm and the human body, as shown in Figure 5a. Additionally, subjective questionnaires were designed to collect comfort evaluations in two parts during the experiment. Firstly, subjective evaluations of specific angles of comfort during the dual-arm transfer and handling process were collected, with a rating scale from 1 (most comfortable) to 10 (least comfortable) and a rating interval of 1. Secondly, after the dual-arm transfer and handling process, ratings were collected to assess how various forces and moments affected the comfort of the human body, with a scale ranging from 1 (minimal impact) to 10 (maximal impact), and a rating interval of 1.

During the transfer and handling process, the hip joint is in a suspended state, requiring stretching of the back and thigh muscles to maintain the stability of the hip joint position. At the same time, the robotic arm directly contacts the back and thighs of the human body, exerting compression on the muscles. Therefore, in this experiment, the surface EMG signals of the back and thigh muscles were mainly measured. The erector spinae, adductor, and tensor fasciae latae muscles, which are major muscles used by the human body to extend the hip joint and support the back and thighs, were selected for surface EMG measurement. The surface EMG signals can reflect the level of muscle fatigue in the human body, thus validating the comfort level through the magnitude of the surface EMG signals. Electrodes were attached to the erector spinae, adductor, and tensor fasciae latae muscles, and connected to the EMG sensor for surface EMG signal acquisition, as shown in Figure 5b,c.

**Figure 6:**
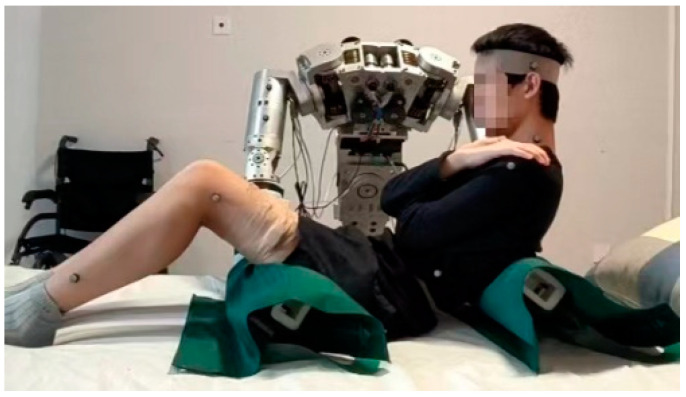
Initial posture for the experiment.

Before the experiment, the robotic arm was adjusted to maintain a horizontal position, and the distance between the robotic arms was recorded. The human body was positioned in the initial posture lying on the nursing bed, as shown in Figure 6. The back of the human body was slightly tilted backward, the knees were raised, and the buttocks maintained contact with the nursing bed while the feet also remained in contact with the bed.

**Figure 7:**
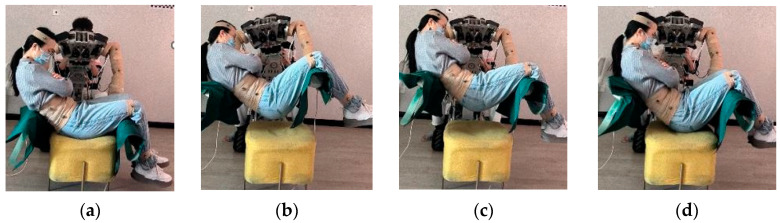
Sequential human movements in the bimanual handling comfort experiment: (**a**) Initiation posture (**b**) Lifting phase (**c**) Posture adjustment phase (**d**) Lowering phase.

During the experimental proceedings, the subject was kept in an initial posture, while adjustments were effectuated to the robotic base. The robot’s left arm was maneuvered to establish contact with the participant’s dorsal region, and the right arm interfaced with the thigh. Subsequently, the subject was gently elevated from the care bed, during which the robotic waist joint was modulated to hoist the subject to a pre-specified elevation. Further, the robotic hip joint was subtly adjusted, engendering a difference in elevation between the left and right robotic arms. This facilitated a gradual and controlled modification of the subject’s posture. By maintaining variances in the elevations of the robotic arms in contact with the subject’s dorsal and leg regions, the subject’s head was manipulated into multiple orientations, including significantly above, slightly above, parallel with, slightly below, and significantly below the level of the legs, as shown in Figure 7. Throughout this process, at each five-degree rotation of the robot, an assessment of the subject’s comfort level was undertaken. A comprehensive subjective evaluation was rendered, employing a score range from 1 to 10. As the perceived level of comfort diminished, the evaluative score increased proportionally.

Additionally, the process including the subtle rotational movement of the subject, facilitated by the manipulation of the robotic arms, was reiterated. Post-rotation, the robotic arms were recalibrated to a level position. The robotic base and waist joint were then mobilized to gently lower the subject back onto the care bed. This experimental process, involving alterations to the inter-arm distance of the robotic apparatus, was repeatedly executed to amass a diverse set of experimental data. Upon the culmination of the experimental process, the subject was immediately solicited for a subjective evaluation of the influence of various forces and torques on comfort levels. The effect score spanned from 1 to 15, with an increase in score commensurate with the escalation in perceived discomfort.

## 3. Results and Discussion

The purpose of the analysis was to validate the real-time solution methodology for the human–robot static model and the comfort assessment function, which necessitated a detailed examination of the data gathered during the experiment. A total of 35 sets of data comprising position, pressure, and surface electromyography information were collected. Initial data processing was focused on the acquired positional information.

**Figure 8:**
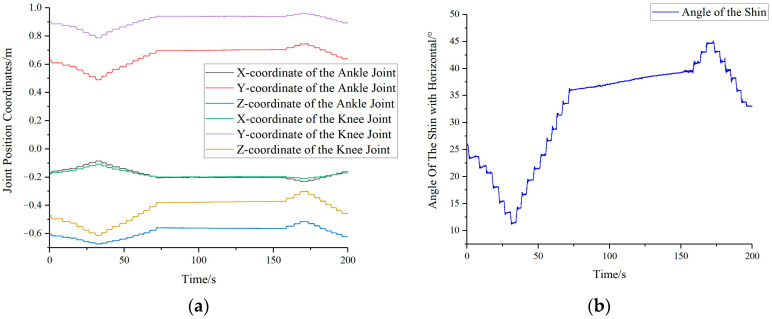
Variations in shin coordinates and their angular relationship with the horizontal plane: (**a**) Transformation in ankle and knee joint coordinates (**b**) Change in the angle between shin and horizontal plane.

By attaching marker points to seven key positions on the human body and capturing the data using the Optitrack motion capture system, real-time coordinate information (*X_1_*, *Y_1_*, *Z_1_*, …, *X_7_*, *Y_7_*, *Z_7_*) for these seven key positions can be obtained. Taking the angle between the shin and the horizontal plane as an example, through motion capture, we can obtain the position information (*X_1_*, *Y_1_*, *Z_1_*, *X_2_*, *Y_2_*, *Z_2_*) of the ankle joint *A_1_* and the knee joint *A_2_*, as shown in Figure 8a. By substituting the above information into Equation (7), we can calculate the real-time angle information (*β_1_*) between the shin and the horizontal plane, as illustrated in Figure 8b.

**Figure 9:**
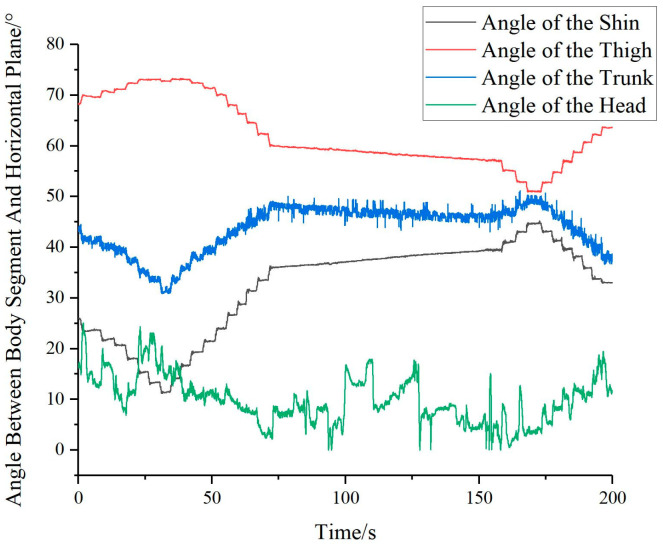
Angular relationship of human shin, thigh, trunk, and head with the horizontal plane.

In a similar fashion, the angles between the human shin, thigh, trunk, and head with the horizontal plane are illustrated in Figure 9.

**Figure 10:**
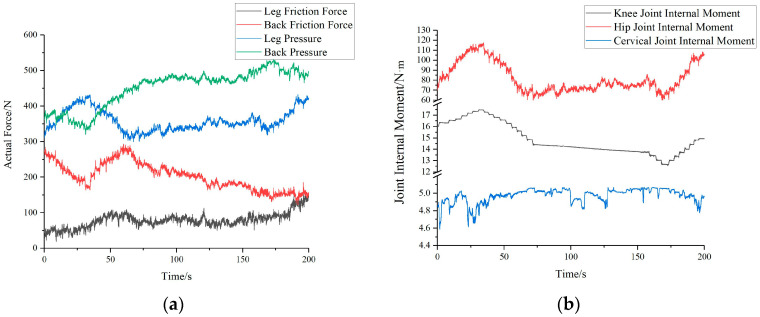
Real-time interaction forces and internal torques between human and robot: (**a**) Real-time interaction force between human and robot (**b**) Human internal torque.

At this point, Tekscan pressure sensors are placed between the human and the robot. These pressure sensors have pressure-sensitive resistors, which exhibit a proportional decrease in resistance as pressure increases. By utilizing the internal scanning circuitry of the pressure sensors, pressure information at the pressure-sensitive points can be obtained, thereby allowing the determination of the pressure *F_1_* between the human back and the robot. Additionally, the angles *β_1_*, *β_2_*, *β_3_*, and *β_4_* between the human shins, thighs, trunk, and head with respect to the horizontal plane and the distance between the robotic arm and the hip joint are known through the use of the Optitrack motion capture system and relevant calculations. At this stage, there are six unknowns, the pressure *F_2_* between the human legs and the robotic arm, the frictional forces *f_1_* and *f_2_* between the human back and thighs with the robot, and the internal moments *M_1_*, *M_2_*, and *M_3_* at the human knee, hip, and neck joints, respectively. By substituting the known values into Equations (1)–(6), real-time interaction forces and internal moments between the human and the robot can be determined, as illustrated in Figure 10a,b.

Through an analysis of the simulation results from the robot transfer process and questionnaire data from the transfer experiments, the majority of the subjects indicated that during the experiment, the influence of the hip joint internal torque on human comfort was greater than that of the normal force, friction force, and the internal torques of the knee and neck joints. Furthermore, the difference in their impact on comfort was quite significant. Thus, different values were assigned to the coefficients ω of each evaluation index to give different weights to each component.

**Figure 11:**
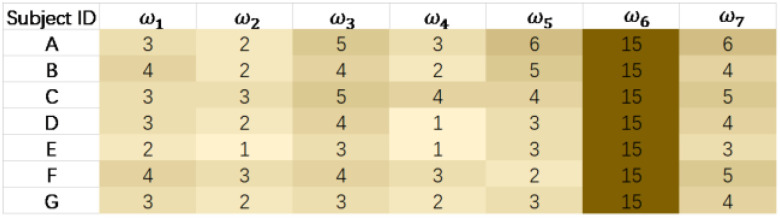
Results from the comfort weight questionnaire.

The results from the comfort weight questionnaire are shown in Figure 11. It can be seen from the survey results that all seven subjects believed that the hip joint torque had a greater effect on comfort. In addition, subjects felt that the impact of hip joint torque on comfort was greater than that of the knee and neck joints. The effect of normal and frictional forces on the leg on comfort was considered less than on the back, which aligns with literature suggesting that the back is more sensitive than the legs [21]. Therefore, the parameter *ω_6_* for the hip joint was taken as 15, while the other parameters *ω_i_* were obtained by averaging the results of the comfort weight questionnaire for the seven subjects. Consequently, the set *ω* was determined to be [3, 2, 4, 2, 4, 15, 4].

**Figure 12:**
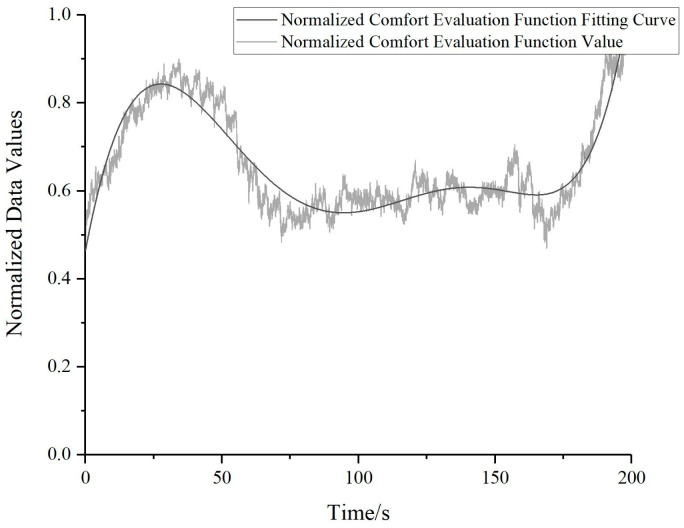
Normalized comfort evaluation function.

Upon determining the parameter values for the comfort evaluation function, these parameters *n* = [1, 1, 1, 1, 1, 1, 1] and *ω* = [3, 2, 4, 2, 4, 15, 4] were integrated into the comfort evaluation function, as in Equation (11). By performing these calculations, the comfort evaluation value for each instant can be obtained, as demonstrated in Figure 12. With time as the horizontal axis, the transparent black line represents real-time comfort evaluation function values that have undergone normalization, while the solid black curve depicts the fitted normalized comfort evaluation function curve.

Surface electromyographic signals of the erector spinae, adductor, and tensor fasciae latae muscles were captured using a Noraxon electromyography sensor. Given that the preservation of hip joint posture during the transfer process necessitates a collective effort of back and leg muscles, a balanced 50% of the electromyographic data was drawn from each of these muscle groups. Specifically, data derived from the erector spinae accounted for the back muscle electromyographic information, whereas data derived from the adductor and tensor fasciae latae represented the leg muscles’ electromyographic information. As a result, an amalgamated electromyographic signal value was generated, consisting of 50% from the erector spinae, and 25% from both the adductor and tensor fasciae latae muscles.

**Figure 13:**
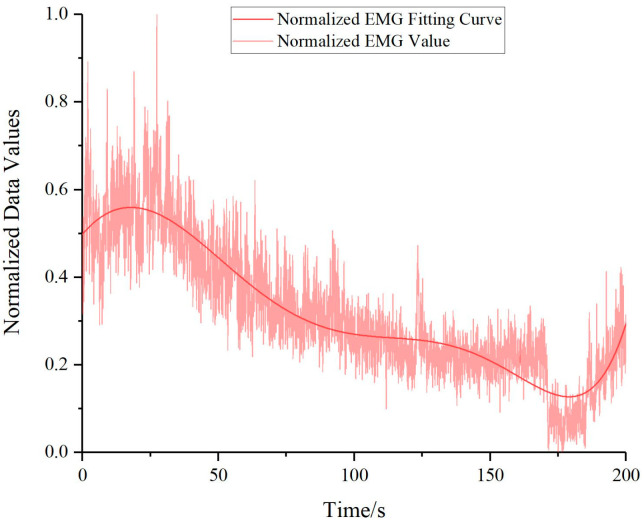
Normalized combined electromyography signal values for erector spinae, adductor, and tensor fasciae latae muscles.

The sEMG data obtained through the Noraxon surface electromyography sensors were subjected to rectification, filtration, and mean feature extraction, resulting in sEMG information that represents muscle comfort. However, at this point, the unit of the sEMG information is measured in microvolts (μV), while the units of the normalized comfort evaluation function values and their fitting curves are dimensionless (“1”), as the unit of each term in Equation (11) has been canceled out to “1”. To facilitate a better comparison of sEMG information with comfort evaluation consistency, we conducted a normalization process on the sEMG data, which transformed the unit of the sEMG information into dimensionless (“1”) after normalization. The results of which are portrayed in Figure 13.

**Figure 14:**
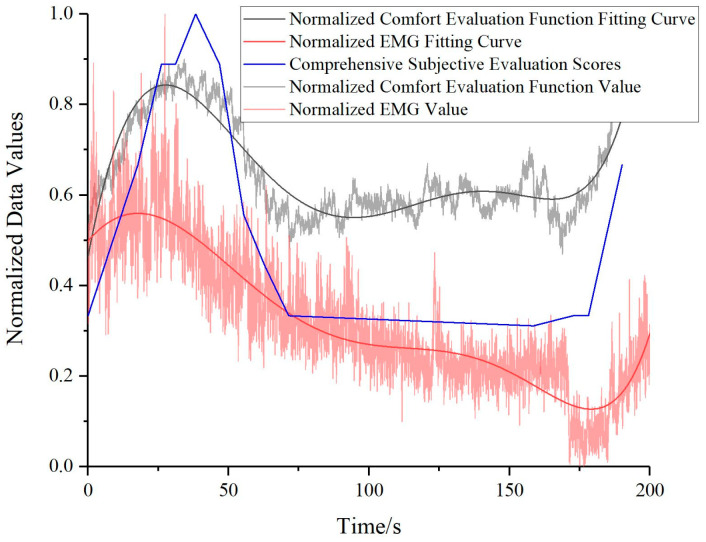
Comparative validation of normalized human comfort evaluation function values, normalized subjective evaluation scores, and normalized electromyographic signal values.

The comfort evaluation function values, the comprehensive subjective questionnaire evaluation scores, and the electromyographic signal values, all having undergone normalization procedures, are juxtaposed for comparison, as depicted in Figure 14. Evidently, the subjective assessment scores correspond closely with the comfort evaluation function values. Moreover, a similar trend is discernible between the comfort evaluation function and the fluctuations in the sEMG signals, although a more in-depth analysis is requisite for discerning the subtleties of these trend variations.

**Figure 15:**
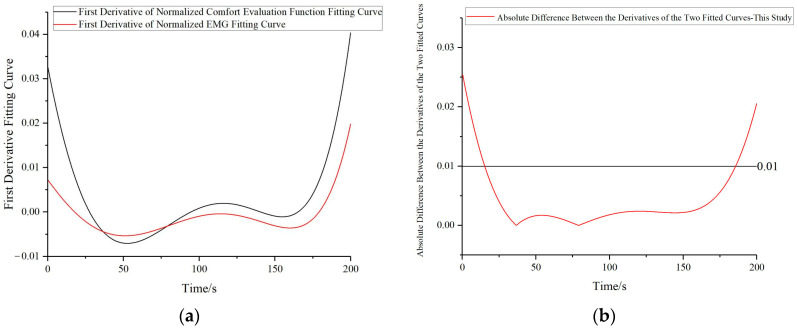
Comparison of trend patterns between human comfort evaluation function and electromyography signal curves: (**a**) Comparison of the first order derivatives of the two fitted curves (**b**) Absolute difference between the derivatives of the two fitted curves.

To provide an objective assessment of the proposed method, we performed curve fitting on the comfort evaluation function values and sEMG information and compared their trends by taking the first derivative, the results are illustrated in Figure 15a. There is a marked concurrence in the trends emerging from both derivative curves. Subsequently, the disparities between the derivatives of the two fitted curves are computed. Specifically, after obtaining the first derivative curves of the two fitted curves, 1000 points were extracted separately from each first derivative curve corresponding to the same abscissa values for both curves. Subsequently, the absolute differences between the corresponding points were calculated, resulting in 1000 data points. Portions wherein the absolute value of the difference falls under 0.01 suggest minor variations in the trends, whereas segments exceeding 0.01 indicate considerable divergences in trends, as showcased in Figure 15b. Hence, it can be deduced that an agreement of 85.1% exists between the trends of the two curves.

**Figure 16:**
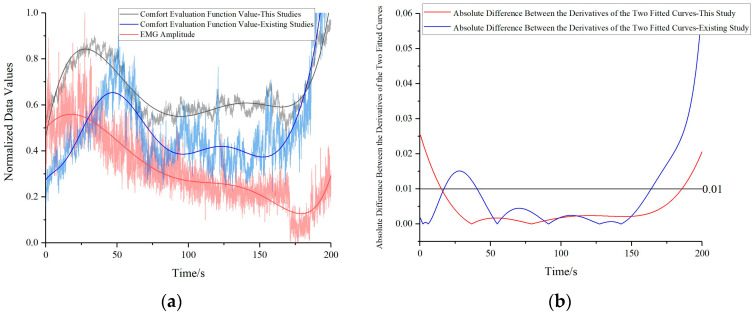
Validation of human comfort evaluation function values for two different methods against electromyography signals: (**a**) Comparison among three curves (**b**) Absolute difference between the derivatives of the three fitted curves.

Comparisons between the normalized comfort evaluation methodology proposed in this study and that presented in existing research [13], yield two curves depicting normalized comfort evaluations and normalized electromyography signal patterns, as demonstrated in Figure 16a. Likewise, first-order derivatives are computed for the fitted curves derived from both the comfort evaluation function values and EMG signal values in this study, as well as those presented in existing research. Absolute differences between these derivatives are calculated and presented in Figure 16b. The trends in the existing research concur at a rate of 70.0%, whereas those in this study display a notably higher agreement rate of 85.1%. Thus, the accuracy of the statics solution methodology and comfort evaluation methodology proposed in this study is effectively substantiated.

This study proposed a mechanics model solving method based on real-time position and pressure information to address the problem of insolubility in the human–robot mechanical model during human transfer processes. By introducing the comfort evaluation function and validating it through subjective questionnaires and sEMG information, we have established a reliable method for assessing human transfer comfort. This evaluation method exhibits objectivity and accuracy, avoiding the randomness associated with fully subjective evaluations and enhancing the reliability of the comfort assessment. Furthermore, this method lays the foundation for ensuring human comfort in human–robot interactions, thereby possessing promising potential in care robot applications.

## 4. Conclusions

This study addresses the challenge of quantifying real-time forces and internal torques experienced by a human body during transfer operations, which traditionally presents as an unsolvable issue. We introduced real-time pressure and position information to solve the biomechanical model dynamically, thereby determining the forces and torques involved in human–robot interactions. Furthermore, this study leveraged a care robot platform to conduct transfer operations aimed at evaluating comfort levels. We developed a comfort evaluation function based on the analysis of questionnaires filled out during these experiments. Concurrently, we compared real-time computed comfort evaluation values with sEMG signals collected during the experiment. The findings validate the effectiveness of our real-time solution approach for the human–robot statics model and the comfort evaluation methodology at a concurrence rate of 85.1%. This marks an improvement over the 70.0% concurrence rate achieved in existing studies, thereby demonstrating an enhanced level of accuracy.

This study highlights two primary innovations. Firstly, to tackle the issue of the human–robot mechanical model as being intractable, we employed a real-time calculation approach based on position and pressure information to determine the human body’s posture, human–robot interaction forces, and internal moments. Secondly, we proposed a real-time comfort evaluation method for human transfer and transportation and verified the accuracy of this evaluation method through both subjective questionnaires and objective sEMG information. However, we acknowledge certain limitations in our research. For the simplification of the human body, we adopted a common approach in biomechanical analysis by representing the human body as a four-bar linkage model, which may introduce some errors in calculating the internal moments, deviating from 100% accuracy.

## Figures and Tables

**Table 1:** Parameters of the Brawne–Fisher human body model segments [19].

Human Segment	Head	Trunk	Upper Arm	Forearm	Hand	Thigh	Shin	Foot
Relative Weight	0.07	0.42	0.07	0.05	0.02	0.23	0.11	0.03
Centroid Radius	#	0.44	0.47	0.42	##	0.44	0.42	0.44

**Table 2:** Parameters of the four-link human body model based on the Brawne–Fisher model.

Human Segment	Head Link	Trunk Link	Thigh Link	Shin Link
Relative Weight	0.07	0.56	0.23	0.14
Centroid Radius	#	0.46	0.44	0.48

**Table 3:** Participant information.

Subject ID	Gender	Height (cm)	Weight (kg)	Shin Length (cm)	Thigh Length (cm)	Trunk Length (cm)	Head and Neck Length (cm)
A	Female	165	49	39	51	49	26
B	Male	180	61	42	55	53	30
C	Female	170	50	40	52	50	28
D	Male	181	60	45	51	55	30
E	Male	183	61	43	56	52	32
F	Female	159	50	35	48	47	29
G	Male	165	51	40	50	46	29

## Data Availability

The dataset analyzed in the current study is available from the corresponding author on reasonable request.
